# Compliance with recommended cancer patient pathway timeframes and choice of treatment differed by cancer type and place of residence among cancer patients in Norway in 2015–2016

**DOI:** 10.1186/s12885-022-09306-9

**Published:** 2022-02-28

**Authors:** Yngvar Nilssen, Odd Terje Brustugun, Morten Tandberg Eriksen, Marianne G. Guren, Erik Skaaheim Haug, Bjørn Naume, Ellen Schlichting, Bjørn Møller

**Affiliations:** 1 Department of Registration, Cancer Registry of Norway, Oslo, Norway; 2 Department of Oncology, Drammen Hospital, Vestre Viken Hospital Trust, Drammen, Norway; 3 Institute of Clinical Medicine, University of Oslo, Oslo, Norway; 4 Division of Surgery, Inflammatory Diseases and Transplantation, Oslo University Hospital, Oslo, Norway; 5 Department of Oncology, Oslo University Hospital, Oslo, Norway; 6 Section of Urology, Vestfold Hospital Trust, Tønsberg, Norway; 7 Institute of Cancer Genomics and Informatics, Oslo University Hospital, Oslo, Norway; 8 Department of Breast and Endocrine Surgery, Department of Cancer, Oslo University Hospital, Oslo, Norway

**Keywords:** Cancer patient pathways, Colorectal cancer, Lung cancer, Breast cancer, Prostate cancer, Waiting time

## Abstract

**Background:**

Cancer patient pathways (CPPs) were implemented in Norway to reduce unnecessary waiting times, regional variations, and to increase the predictability of cancer care for the patients. This study aimed to determine if 70% of cancer patients started treatment within the recommended time frames, and to identify potential delays.

**Methods:**

Patients registered with a colorectal, lung, breast, or prostate cancer diagnosis at the Cancer Registry of Norway in 2015–2016 were linked with the Norwegian Patient Registry and Statistics Norway. Adjusting for sociodemographic variables, multivariable quantile (median) regressions were used to examine the association between place of residence and median time to start of examination, treatment decision, and start of treatment.

**Results:**

The study included 20 668 patients. The proportions of patients who went through the CPP within the recommended time frames were highest among colon (84%) and breast (76%) cancer patients who underwent surgery and lung cancer patients who started systemic anticancer treatment (76%), and lowest for prostate cancer patients who underwent surgery (43%). The time from treatment decision to start of treatment was the main source of delay for all cancers. Travelling outside the resident health trust prolonged waiting time and was associated with a reduced odds of receiving surgery and radiotherapy for lung and rectal cancer patients, respectively.

**Conclusions:**

Achievement of national recommendations of the CCP times differed by cancer type and treatment. Identified bottlenecks in the pathway should be targeted to decrease waiting times. Further, CPP guidelines should be re-examined to determine their ongoing relevance.

**Supplementary Information:**

The online version contains supplementary material available at 10.1186/s12885-022-09306-9.

## Introduction

Colorectal, lung, breast, and prostate cancer are the four most common cancer types in Norway with 16 279 new cases diagnosed in 2020, which represents 45.8% of all new cancers [1]. The incidence and prognosis vary substantially between these cancer types [1, 2].

The time it takes for a patient to be diagnosed, undergo necessary staging procedures, and to start treatment may affect their prognosis since detecting a cancer at an earlier stage increases the likelihood of having curative treatment options [3]. In addition, the uncertainty associated with a prolonged time to diagnosis and start of treatment may cause psychological distress for the patients which was especially in focus when Norway implemented cancer patient pathways (CPP) in all health trusts in 2015, following the Danish model. Each health trust was responsible for actioning CPPs in their region. Unlike the Danish model that also focused on improving survival, the primary aims of the Norwegian CPPs were to reduce unnecessary waiting times and regional variation, and to increase the predictability of the cancer care for patients and their relatives [4, 5]. The CPPs for colorectal, lung, breast, and prostate cancer were implemented in January 2015, while 24 other CPPs were implemented throughout the year [6–10]. A previous study showed a significant decreasing trend in waiting time to surgery and radiotherapy for colorectal, lung, breast and prostate cancer in Norway [11].

A patient can be referred to a CPP by a general practitioner (GP), a specialist, a private imaging centre or from within a hospital if there is a “reasonable suspicion of cancer” based on the patient’s symptoms and signs. With the implementation of CPPs, the new role of a cancer pathway coordinator was introduced to help manage referrals, plan appointments, and provide information to the patients until the first specialist consultation. Different to the extra funding that was allocated to implement CPPs in Denmark, the implementation of CPPs in Norway did not receive any additional government funding. Like the Danish model, the CPPs include guidance for recommended diagnostic procedures and recommendations for maximum days from when the hospital receives the referral to the first patient visit to the specialist, the specialist visit to a treatment decision, the treatment decision to the start of treatment, and the overall number of days from when the hospital receives the referral to the start of treatment. These time frames are individually determined for each cancer type.

The Norwegian Directorate of Health set a national target that at least 70% of all new cancer patients should be included in a CPP, and that at least 70% of these should be treated within the defined time frames [7–10]. The target of 70% was not supposed to be a minimum where 100% is optimal, but rather that 70–80% of patients could be examined, diagnosed and start treatment in a standardised way, whereas the remaining patients would require more thorough examination and deliberation before starting treatment. A previous study showed that over 70% of all colorectal, lung, breast and prostate cancer patients were included in a CPP during 2015–2016 [12]. However the monitoring of the CPP data by the Norwegian Directorate of Health has reported regional variation in the proportion of patients starting their treatment within the given time frames [13]. Regional variation in type of treatment administered was also observed prior to CPPs as reported in two recent Norwegian studies [14, 15]. These showed that patients living in health trusts without a treating unit or who had to travel had a lower odds of receiving that specific treatment.

The present study describes colorectal, lung, breast, and prostate cancer patients diagnosed and included in a CPP in Norway in 2015–2016. The aims were to determine the proportion who started treatment within the recommended time frames and to identify differences in waiting time within the CPPs. Additionally, the study examined whether travelling for treatment outside of the resident health trust affected the choice of treatment and waiting time.

## Methods

### Cancer registry of Norway

Since 1952, it has been mandatory for all hospitals, pathology laboratories and GPs in Norway to report all newly diagnosed malignant disease to the Cancer Registry of Norway (CRN). The CRN also receives death certificates for all patients with a cancer diagnosis from the Cause of Death Registry. Using the personal identification number assigned to all Norwegian citizens since 1964, the CRN is linked monthly with the National Population Register to update vital status (death or emigration), and three times per year with the Norwegian Patient Registry (NPR) to ensure completeness of cancer cases. The quality, comparability, completeness, validity, and timeliness of the data in the CRN have been evaluated to be high, with an estimated completeness of 98.8% for all cancer sites together [16].

### Norwegian patient registry

The NPR is a national health register that holds data on all patient visits to government-funded hospitals in Norway. Reporting to the NPR is mandatory, and its database covers over 99% of all patient visits to specialised health care services [17]. The NPR database includes data regarding patients’ comorbidity index and information about who are included in a CPP, what treatment they received and their event dates.

### Statistics Norway

The national statistics institute, Statistics Norway, holds individual-level information in areas such as population, health, finance and education for the entire Norwegian population. Education data have been collected from various national databases since 1970. The tax authorities provide Statistics Norway with personal and household income data, which are available since 1967 and 1993, respectively, while information about type of household is available from 2004 onwards. Information about the patient’s socioeconomic status (SES) was measured through household income and education.

### Data linkage

**Fig. 1:**
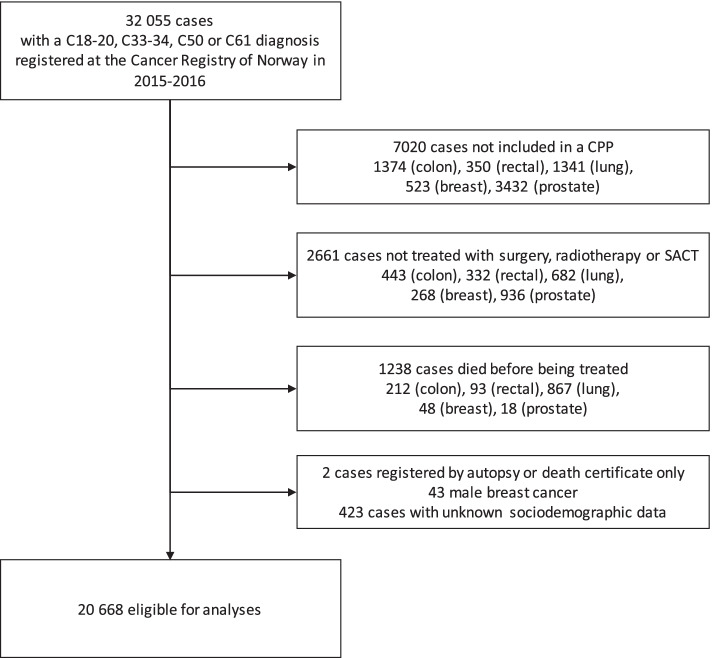
Patient selection diagram

The CRN data were linked with the NPR and Statistics Norway data using the unique 11-digit personal identification number assigned to every Norwegian resident. The study population included all patients with a colorectal (ICD-10 code C18-20), lung (ICD-10 code C33-34), breast (ICD-10 code C50), or prostate (ICD-10 code C61) cancer diagnosis registered at the CRN between 1 January 2015 and 31 December 2016. Patients who were not included in a CPP, those who were not treated with one of surgery, radiotherapy, systemic anticancer therapy (SACT) or active surveillance (prostate only) within one year of diagnosis, and those with unknown SES, were excluded. Cases who were registered by either autopsy or death certificate alone and male breast cancer patients (due to an expected low number of cases for the analyses) were excluded (Fig. 1).

### Treatment modalities

The treatment modality, as obtained from the CPP data from the NPR, was surgery of a primary tumour, radiotherapy (curative or palliative), SACT, or active surveillance (prostate only). SACT included chemotherapy, targeted therapy, immunotherapy, hormonal therapy or any other public hospital-administered anti-cancer medication. If a patient underwent several treatment modalities, the first occurrence, e.g. neoadjuvant treatment, was registered as the treatment.

### Cancer patient pathways

The following data are registered in the CPP: (1) date the referral is received by the hospital (start CPP), (2) date the patient attends the hospital (outpatient, hospitalisation, imaging) or specialist, and the clinical examination starts (start examination), (3) date when the treatment decision is made (treatment decision), and finally (4) the date the patient begins treatment (start treatment). If patients were transferred between hospitals, the treatment decision date registered at the first hospital was used. For prostate cancer patients undergoing active surveillance the start of treatment will be the day the doctor speaks with the patient. The Norwegian Directorate of Health has defined a set of organ-specific pathway time indicators (OF). These contain the recommended maximum time from start CPP to start examination (OF1), time from start examination to treatment decision (OF2), time from treatment decision to start treatment (OF3), and time from start CPP to start treatment (OF4). These timeframes are individually defined by cancer and treatment type (Table 1).

**Table 1:** The recommended maximum number of days from the referral is received in the specialist health care to the patients attend the hospital for the first time (OF1), from start examination to treatment decision (OF2), from treatment decision to start treatment (OF3), and from start CPP to start treatment (OF4) for colon, rectal, lung breast and prostate cancer

	**Cancer type**				
OF	**Colon**	**Rectal**	**Lung**	**Breast**	**Prostate**
1	9	9	7	7	10
2	12	12	21	7	24
3-S	14	14	14	13	32
3-RT	18	18	14	NA	32
3-SACT	14	14	7	10	3
3-AS	NA	NA	NA	NA	3
4-S	35	35	42	27	66
4-RT	39	39	42	NA	66
4-SACT	35	35	35	24	37
4-AS	NA	NA	NA	NA	37

*Abbreviations*: *S* Surgery, *RT* Radiotherapy, *SACT* Systemic anticancer treatment, *AS* Active Surveillance, *NA* Not applicable, *OF* Organ-specific pathway time indicators

### Stage and risk groups

Stage of disease was categorised as localised, regional, metastatic, or unknown for colorectal, lung and prostate cancer [18]. Local and locally advanced prostate cancer patients were additionally categorised as low-risk, intermediate-risk, high-risk localised, and high-risk locally advanced as defined by the 2012 European Association of Urology (EAU) guidelines [19]. Risk group information is dependent on the reporting of cT and PSA level by the clinicians together with grade from the pathologist, and these data were known for 85% of the patients in 2019 [20]. Those without a risk-group classification were either metastatic or unknown. For breast cancer, stage was classified as stage I, II, III, IV or unknown. Stage IV breast cancer may include synchronous, but not metachronous tumours as only notifications received within the diagnostic period of four months were used [21].

### Geography

Norway consists of 19 health trusts and two privately funded hospitals that are responsible for specialised cancer care in their catchment areas. These 21 will be referred to as “health trusts” in this study. A patient’s health trust was based on their place of residence at the time of diagnosis, independent of where they were diagnosed or treated. A dichotomous variable “Treatment hospital in health trust” was defined as “Yes” if there was a hospital performing the treatment within the patient’s health trust and “No” if otherwise. This categorisation is specific to each cancer type and treatment modality (Supplementary Table 1).

### Statistical analysis

The waiting times are presented as standard statistical measures such as proportions, median, interquartile intervals, and ranges. Although colon and rectal cancer patients share almost the same CPP, their individual treatment regimens differ, therefore all analyses were performed for colon and rectal cancer separately. Multivariable quantile (median) regressions, with time in OF1, OF2, OF3 (surgery, radiotherapy and SACT) and OF4 (surgery, radiotherapy and SACT) as the dependent variables, were performed for each cancer site and adjusted for case-mix, i.e., year of diagnosis, age group and stage at diagnosis, sex, income group, marital status, comorbidity index and health trust. For prostate cancer, two regression models were analysed (OF3 and OF4) including active surveillance as a treatment option. The definition and derivation of income and comorbidity have been described elsewhere [12]. To analyse whether having a treating hospital within the patient’s health trust is associated with waiting times and choice of treatment (surgery or radiotherapy), multivariable quantile and logistic regressions were performed for cancer type (rectum, lung and prostate) and treatment modality, adjusting for year of diagnosis, age group, sex, stage and treatment hospital in health trust. This analysis was not performed for breast and colon cancer as patients are predominantly treated within their health trust. The age groups 80–89 and 90 + were combined in the analysis for prostate cancer to achieve convergence. Wald test was used to assess the significance of the different explanatory variables. A *p*-value < 0.05 was considered significant. The statistical program Stata 16.1 was used for all analyses [22].

## Results

### Study population

Between 1 January 2015 and 31 December 2016, 32 055 cases were identified with a primary colorectal, lung, breast or prostate cancer diagnosis. Of these, 7020 (21.9%) cancer cases were excluded since they were not included in a CPP. This equates to 23.1% of colon cancer cases, 13.3% of rectal cancer cases, 21.8% of lung cancer cases, 7.4% of breast cancer cases and 33.4% of prostate cancer cases. There were 2661 (8.3%) cancer cases (colon: 443, rectal: 332, lung: 682, breast: 268, prostate: 936) who were not treated with one of surgery, radiotherapy, SACT or active surveillance (prostate only) within one year of diagnosis and therefore, these cases were excluded. In addition, 1238 cancer cases (colon: 212, rectal: 93, lung: 867, breast: 48, prostate: 18) who died before receiving any treatment were excluded. Other cases who were registered by either autopsy or death certificate alone (*n* = 2) were excluded. Additionally, male breast cancer cases (*n* = 43) and cases with unknown SES (*n* = 423) were excluded from the analyses. As a result, 20 668 cases were eligible for analyses (Fig. 1). Patient characteristics by cancer type are available in supplementary material (Supplementary tables 2, 3, 4, 5 and 6).

### Colon cancer

**Fig. 2:**
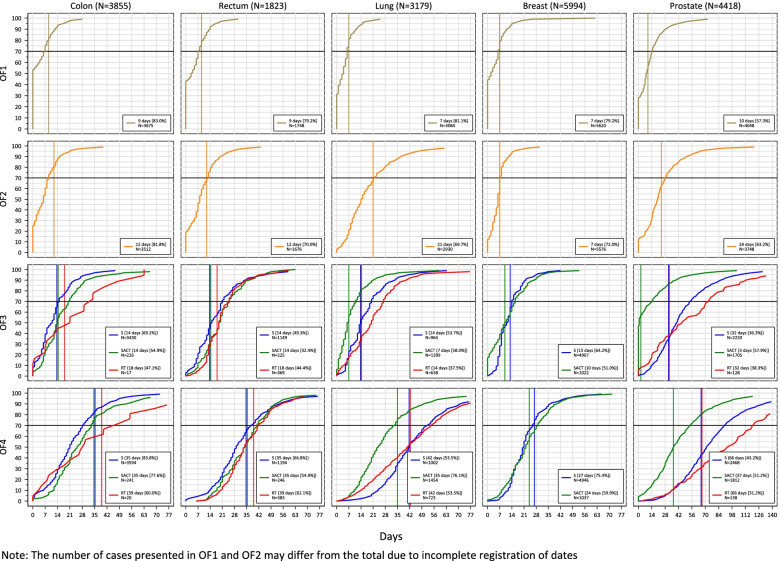
The cumulative proportion of colon, rectal, lung, breast and prostate cancer patients diagnosed in 2015–2016 in Norway who underwent surgery (S), radiotherapy (RT) and systemic anticancer treatment (SACT), by OF1, OF2, OF3 and OF4 in comparison to the recommended timeframes

Of the eligible 3855 colon cancer patients, 93%, 1% and 6% had surgery, radiotherapy and SACT, respectively, as first treatment. In Norway, 84% of colon cancer patients underwent surgery within the recommended 35 days since referral (Fig. 2). 81%, 82% and 65% of patients had waiting times within the recommendations for OF1, OF2 and OF3 (surgery), respectively. Nineteen health trusts were able to start surgery (OF4) for more than 70% of colon cancer patients within 35 days (Supplementary table 7). The remaining two health trusts treated 67–69% of their patients within 35 days. Only one health trust did not succeed in meeting the guidelines for OF1 and four health trusts were unable to meet the guidelines for OF2. Eight health trusts did not have 70% of their patients meet the recommended time frame for OF3 and for these the proportion of patients starting treatment according to the recommendations ranged from 23 to 67% (Supplementary table 7). The regional variation in OF4 remained statistically significant after adjusting for case-mix, with a 25-day difference between the health trust with the shortest and longest waiting time (Supplementary table 12).

### Rectal cancer

**Fig. 3:**
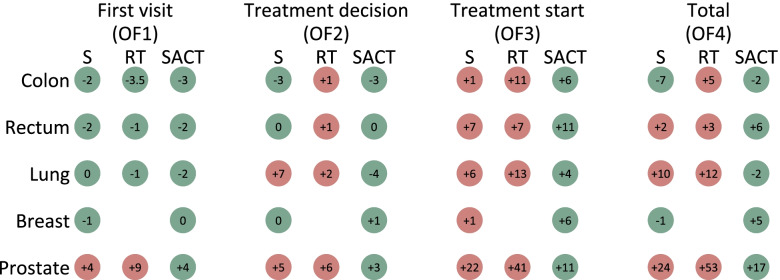
The number of days between the different recommended time frames and the time when 70% passed OF1, OF2, OF3 and OF4 for colon, rectal, lung, breast and prostate cancer patients diagnosed in 2015–2016 in Norway who underwent surgery (S), radiotherapy (RT) and systemic anticancer treatment (SACT)

For rectal cancer, four and nine of the 21 health trusts had a unit performing surgery and radiotherapy, respectively (Supplementary table 1). Of the eligible 1823 rectal cancer patients, the initial treatment was surgery, radiotherapy and SACT for 66%, 21% and 14% respectively. For OF4, 67% and 55% of rectal cancer patients received surgery and SACT within 35 days, respectively, while 62% started treatment with radiotherapy within 39 days (Fig. 2). It took 41 days for 70% of patients who underwent SACT to start treatment (OF4), which was six days longer than the recommended time frame (Fig. 3). The main contributor was the 11-day delay observed in OF3. 76% and 68% of patients had an OF1 and OF2, respectively, lower than the recommended number of days. Only 48%, 43% and 32% of patients who underwent surgery, radiotherapy and SACT, respectively, had waiting times within the recommended guidelines for OF3 (Supplementary table 8).

Only 10 health trusts had more than 70% of patients having surgery (OF4) within 35 days, while five health trusts were able to start radiotherapy (OF4) within 39 days. Over 65% of all patients in all health trusts had an OF1 within the recommended time (Supplementary table 8). Nine health trusts had 42%-66% of patients with an OF2 within 12 days. One health trust exceeded 70% and had 86.4% of their patients receive surgery within 14 days of their treatment decision (OF3). For radiotherapy, another health trust exceeded 70% and had 82% of their patients started treatment within 14 days of the decision. The regional variation in OF4 was statistically significant after adjusting for case-mix (Supplementary table 13).

Of all eligible rectal cancer patients who either received surgery or underwent radiotherapy, 6% and 36% lived in a health trust without a hospital performing surgery or radiotherapy, respectively. These patients had to be referred to another hospital and waited 6 days (Days: 6.0, 95%CI: [2.4,9.6]) and 8 days (Days:8.0, 95%CI: [4.3, 11.7]) longer to surgery and radiotherapy, respectively, compared to those living in a health trust with a hospital performing the treatment (Table 2). The odds of receiving radiotherapy as the initial treatment was 40% lower (OR = 0.59, 95%CI: [0.47, 0.74]) for patients living in a health trust without a hospital performing radiotherapy. No difference was observed for patients receiving surgery (Table 3).

**Table 2:** Univariable and multivariable quantile regressions for OF1, OF2, OF3 and OF4 for rectal, lung and prostate cancer patients diagnosed in 2015–2016 in Norway. Estimates are only presented for the variable “Treatment hospital in health trust”, but the multivariable analyses are additionally adjusted for year of diagnosis, age group, sex and stage. Analyses were stratified for surgery and radiotherapy

		**Rectum**		**Lung**		**Prostate**	
		**Univariate**	**Multivariate**	**Univariate**	**Multivariate**	**Univariate**	**Multivariate**
	**OF**	**Coeff [95%CI]**	**Coeff [95%CI]**	**Coeff [95%CI]**	**Coeff [95%CI]**	**Coeff [95%CI]**	**Coeff [95%CI]**
**Treatment hospital in health trust—surgery**							
Yes	OF1	0.00 [ref]	0.00 [ref]	0.00 [ref]	0.00 [ref]	0.00 [ref]	0.00 [ref]
No	OF1	4.00 [2.53,5.47]	4.00 [2.18,5.82]	-2.00 [-2.64,-1.36]	-2.00 [-2.56,-1.44]	1.00 [-0.20,2.20]	1.00 [-0.09,2.09]
*p*-value	OF1		< 0.001		< 0.001		0.073
**Treatment hospital in health trust—radiotherapy**							
Yes	OF1	0.00 [ref]	0.00 [ref]	0.00 [ref]	0.00 [ref]		
No	OF1	1.00 [-1.09,3.09]	2.00 [-0.01,4.01]	2.00 [0.64,3.36]	2.00 [0.97,3.03]		
*p*-value	OF1		0.051		< 0.001		
**Treatment hospital in health trust—surgery**							
Yes	OF2	0.00 [ref]	0.00 [ref]	0.00 [ref]	0.00 [ref]	0.00 [ref]	0.00 [ref]
No	OF2	-2.00 [-6.91,2.91]	-2.00 [-6.57,2.57]	1.00 [-1.47,3.47]	0.00 [-2.21,2.21]	5.00 [2.83,7.17]	6.00 [4.20,7.80]
*p*-value	OF2		0.391		1.00		< 0.001
**Treatment hospital in health trust—radiotherapy**							
Yes	OF2	0.00 [ref]	0.00 [ref]	0.00 [ref]	0.00 [ref]		
No	OF2	4.00 [1.48,6.52]	4.00 [1.48,6.52]	1.00 [-1.77,3.77]	-0.14 [-2.36,2.07]		
*p*-value	OF2		0.002		0.899		
**Treatment hospital in health trust—surgery**							
Yes	OF3	0.00 [ref]	0.00 [ref]	0.00 [ref]	0.00 [ref]	0.00 [ref]	0.00 [ref]
No	OF3	4.00 [0.82,7.18]	2.00 [-0.69,4.69]	4.00 [2.19,5.81]	4.33 [3.06,5.61]	2.00 [-0.75,4.75]	2.00 [-1.03,5.03]
*p*-value	OF3		0.145		< 0.001		0.196
**Treatment hospital in health trust—radiotherapy**							
Yes	OF3	0.00 [ref]	0.00 [ref]	0.00 [ref]	0.00 [ref]		
No	OF3	1.00 [-1.25,3.25]	3.00 [0.59,5.41]	4.00 [1.59,6.41]	6.00 [3.89,8.11]		
*p*-value	OF3		0.015		< 0.001		
**Treatment hospital in health trust—surgery**							
Yes	OF4	0.00 [ref]	0.00 [ref]	0.00 [ref]	0.00 [ref]	0.00 [ref]	0.00 [ref]
No	OF4	6.00 [1.57,10.43]	6.00 [2.41,9.59]	3.00 [0.59,5.41]	4.50 [2.01,6.99]	6.00 [1.98,10.02]	5.00 [0.87,9.13]
*p*-value	OF4		0.001		< 0.001		0.018
**Treatment hospital in health trust—radiotherapy**							
Yes	OF4	0.00 [ref]	0.00 [ref]	0.00 [ref]	0.00 [ref]		
No	OF4	8.00 [4.18,11.82]	8.00 [4.28,11.72]	4.00 [-0.55,8.55]	5.00 [1.67,8.33]		
*p*-value	OF4		< 0.001		0.003		

This analysis was not performed for breast and colon cancer as patients are predominantly treated within their health trust

**Table 3:** Univariable and multivariable logistic regressions for receiving surgery and radiotherapy for rectal, lung and prostate cancer patients diagnosed in 2015–2016 in Norway. Estimates are only presented for the variable "Treatment hospital in health trust", but the multivariable analyses were additionally adjusted for year of diagnosis, age group, sex and stage

	**Rectum**		**Lung**		**Prostate**	
	**Univariate**	**Multivariate**	**Univariate**	**Multivariate**	**Univariate**	**Multivariate**
	**Odds Ratio [95%CI]**	**Odds Ratio [95%CI]**	**Odds Ratio [95%CI]**	**Odds Ratio [95%CI]**	**Odds Ratio [95%CI]**	**Odds Ratio [95%CI]**
**Treatment hospital in health trust—Surgery**						
Yes	1.00 [ref]	1.00 [ref]	1.00 [ref]	1.00 [ref]	1.00 [ref]	1.00 [ref]
No	1.01 [0.72,1.42]	1.00 [0.69,1.45]	0.75 [0.65,0.86]	0.74 [0.62,0.88]	0.97 [0.87,1.08]	1.12 [0.98,1.27]
*p*-value	0.381	0.570	< 0.001	< 0.001	0.539	0.092
**Treatment hospital in health trust—Radiotherapy**						
Yes	1.00 [ref]	1.00 [ref]	1.00 [ref]	1.00 [ref]		
No	0.60 [0.48,0.76]	0.59 [0.47,0.74]	1.04 [0.88,1.22]	1.06 [0.90,1.24]		
*p*-value	< 0.001	< 0.001	0.653	0.483		

This analysis was not performed for breast and colon cancer as patients are predominantly treated within their health trust

### Lung cancer

Seven and nine of the 21 health trusts had a unit performing surgery and radiotherapy, respectively (Supplementary table 1). Of the eligible 3179 lung cancer patients, 32%, 23% and 46% had initial treatments of surgery, radiotherapy and SACT, respectively. While only 53% of those treated with surgery or radiotherapy started treatment within the recommended 42 days (OF4), 76% were treated with SACT within the recommended 35 days (OF4) (Fig. 2). It took 52 and 54 days for 70% of patients who underwent surgery and radiotherapy, respectively, to start treatment (OF4) (Fig. 3). The main contributor was the six-and 13-day delays observed in OF3. 80% of lung cancer patients had an OF1 of at most seven days, while 68% had an OF2 of a maximum of 21 days. Only 53%, 21% and 58% of patients who underwent surgery, radiotherapy and SACT, respectively, began treatment within the given time frames for OF3 (Supplementary table 9).

For lung cancer patients, only one health trust was able to have at least 70% of their patients start radiotherapy in 42 days, only two health trusts had at least 70% of their patients receiving surgery in 42 days, and 15 health trusts had at least 70% of their patients start SACT in 35 days (Supplementary table 9). Only one health trust started treatment for over 70% of the patients within the time frame in OF4 for all treatment modalities.

All health trusts had over 65% of patients with an OF1 within seven days (Supplementary table 9). The proportion of patients who had an OF2 of at most 21 days ranged from 48.5% to 84.5%, and over 70% was achieved in nine health trusts. The proportion of patients with an OF3 within the time frame ranged from 22.6% to 81.3%, 16.2% to 62.2%, and 20.7% to 76.3% among patients undergoing surgery, radiotherapy and SACT, respectively. Regional variation in OF4 remained statistically significant after adjusting for case-mix, and differences of 20 days (-8.6, 95%CI: [-14.1, -3.1] vs 11.4, 95%CI: [3.5, 19.3]), 29 days (-10.1, 95%CI: [-16.0, -4.3] vs 19.0, 95%CI: [4.0, 33.9]), and 17 days (-5.6, 95%CI: [-9.9, -1.3] vs 11.4, 95%CI: [5.3, 17.5]) were observed between the health trusts with the shortest and longest times to surgery, radiotherapy and SACT, respectively (Supplementary table 14).

Of all eligible lung cancer patients, 54% of all patients treated with surgery and 49% of all patients treated with radiotherapy, lived in a health trust without a hospital performing these treatments. These patients experienced a median 4.5 days (Days: 4.5, 95%CI: [2.0, 7.0]) and 5.0 days (Days: 5.0, 95%CI: [1.7, 8.3]) longer OF4 than patients living in a health trust with a hospital performing these treatments (Table 2). The odds of receiving surgery as the initial treatment was 26% lower (OR = 0.74, 95%CI: [0.62, 0.88]) for patients living in a health trust without a hospital performing surgery. No difference was observed for patients who received radiotherapy (Table 3).

### Breast cancer

Of the eligible 5994 breast cancer patients, 83%, 0.2% and 17% had initial treatment of surgery, radiotherapy and SACT, respectively. The 11 breast cancer patients who were initially treated with radiotherapy were excluded due to low numbers. 76% and 60% of breast cancer patients were treated with surgery and SACT respectively within 27 and 24 days (OF4), respectively (Fig. 2). At 30 days, six days after the OF4 time frame, 70% of patients had started SACT (Fig. 3). 79% of patients had an OF1 and 72% had an OF2 within recommended days. While 64% of patients were treated with surgery within 13 days of a decision, only 51% started SACT within 10 days (Supplementary table 10).

In sixteen health trusts 70% or more of patients underwent surgery within 27 days (OF4). The remaining health trusts ranged from 41 to 67% (Supplementary table 10). For SACT, only six health trusts managed to achieve the target of 70% starting treatment within 24 days (OF4). Patients in seven health trusts did not meet recommended OF1 and OF2 time frames. Between 38 and 97% of patients underwent surgery and between 35 and 78% of patients started SACT within recommended OF3 time frames. The regional variation in OF4 remained statistically significant after adjusting for case-mix and there was a 16-day (-5.4, 95%CI: [-8.1, -2.8] vs 9.7, 95%CI: [7.4, 12.0]) and a 14-day (-9.5, 95%CI: [-13.8, -5.1] vs 5.0, 95%CI: [2.7, 7.3]) difference between the health trust with the shortest and longest time to surgery and SACT, respectively (Supplementary table 15).

### Prostate cancer

Twelve of the 21 health trusts had a unit performing surgery (Supplementary table 1). Of the eligible 5817 prostate cancer patients, the initial treatment was surgery, radiotherapy, SACT and active surveillance for 42%, 2%, 31% and 24%, respectively. The overall recommended waiting times from referral to treatment (OF4) were achieved for 43% of surgery patients and 50% of SACT patients, and it took an additional 24 and 17 days for 70% of patients to have surgery and start SACT, respectively (Fig. 2, Fig. 3). Only 36% and 56% met the recommended time frames from treatment decision to start of surgery and SACT (OF3), respectively (Fig. 3, Supplementary Fig. 1). Of patients who underwent active surveillance, only 54% had a treatment decision (OF2) within the recommended time frame of 24 days (data not shown).

None of the health trusts were able to meet the target of 70% of patients receiving surgery within 66 days (OF4). Their proportions ranged from 8 to 68% (Supplementary table 11). Three health trusts managed to start treating at least 70% of patients with SACT within 37 days (OF4). Six health trusts achieved the recommended OF1 time frame, while only four health trusts achieved the OF2 target. For OF3, only one health trust met the target for surgery and two health trusts met the target for SACT. Regional variation in OF4 for all treatment modalities remained statistically significant after adjusting for case-mix (Supplementary table 16). There was a 66-day and 47-day difference between the health trust with the shortest and longest times to surgery and SACT, respectively (Supplementary table 16).

Of all prostate cancer patients who underwent surgery, 28% were living in a health trust without a hospital performing surgery. These patients had an OF4 that was 5 days (Days: 5.0, 95%CI: [0.9,9.1]) longer, compared to those living in a health trust with a hospital performing surgery (Table 2).

## Discussion

The national target that over 70% of all cancer patients included in a CPP should be treated within the recommended time frames was achieved among colon and breast cancer patients who had surgery, and lung cancer patients who started SACT. With the exception of prostate cancer, over 70% of all colorectal, lung and breast cancer patients started examination and had a treatment decision within the recommended time frames. Overall, a patient’s place of residence was associated with longer waiting times to treatment and differences in treatment selection for rectal and lung cancer patients.

### Colon cancer

In 19 of 21 health trusts, more than 70% of colon cancer patients had their surgery within 35 days of entering the CPP. The Norwegian Colorectal Cancer Registry reported over 40 hospitals performing surgery for colon cancer in Norway in 2019 [23]. Due to the wide distribution of treating hospitals around the country, it is likely that most colon cancer patients received surgery within their health trust and did not require a referral which would increase waiting times.

### Rectal cancer

Less than 70% of rectal cancer patients received surgery, radiotherapy or SACT within the recommended time frames (OF4). While colon and rectal cancer share identical time frames in a common CPP, pre-decision work-up is more complex for rectal cancer patients due to rigid proctoscopy and pelvic MRI [24]. In addition, patients who live in health trusts that do not have a treating hospital are referred to the regional hospital, which may explain prolonged waiting times. Patients who are candidates for pre-operative radiotherapy may be examined locally and then referred to the regional hospital with radiotherapy for curative treatment, where they are discussed at the multidisciplinary team (MDT) meeting [25]. If the decision of initial treatment is surgery, patients are referred back to their local hospital. If the decision is pre-operative radiotherapy, treatment is provided at the regional radiotherapy centre. Patients receiving SACT with curative intent include those with locally advanced primary tumours and those with resectable synchronous metastases in addition to primary tumours; these patients require thorough examination including discussions at both rectal and liver cancer MDT-meetings, which may also increase waiting times. Patients with metastatic disease usually start life-prolonging SACT at the local hospital, and thus the waiting time may be minimal.

For rectal cancer patients who received surgery or radiotherapy, those who did not live in a health trust with a treating hospital waited a week longer than those who did, and patients not living in a health trust with a radiotherapy unit were 40% less likely to receive radiotherapy. These radiotherapy results are consistent with a previous Norwegian study that found that the use of preoperative radiotherapy was 50% higher in patients from a local hospital performing radiotherapy compared to those from a hospital without radiotherapy services [26].

### Lung cancer

The recommended time frames were only met for lung cancer patients treated with SACT. The patients receiving SACT have a disease that has often progressed beyond a local tumour, making the diagnostic examination easier and less time consuming. In addition, SACT is performed locally where there is good capacity and short waiting times. Of the patients who underwent surgery or radiotherapy, 70% started treatment within 1.5 week after the recommended time frame. For some patients, the effects on prognosis of such a delay is likely to be seen as negligible, however, for patients who experience rapid progression of the disease, these days could mean the difference between operable and inoperable, in addition to the already severe psychological stress they are experiencing. These delays may be caused by low capacity in the hospitals performing surgery and radiotherapy. In addition, the large variation of overall waiting time between health trusts may reflect different clinical practices and availability of diagnostic tools, e.g. PET-CT machines.

For lung cancer patients who received surgery or radiotherapy, those who did not live in a health trust with a treating hospital waited longer than those who did, and patients not living in a health trust with a surgical unit were 26% less likely to receive surgery. Transferring between health trusts for treatment may have contributed to the delays since referrals between hospitals were sent by hardcopy and subject to postal service. The lack of electronic transmission of referrals and documentation has been identified and solutions are gradually being implemented.

### Breast cancer

While over 70% of breast cancer patients who underwent surgery were treated within the recommended time frame of 27 days, regional differences existed. Due to a lack of breast radiologists, some breast diagnostic centres may struggle with long waiting times from the start of examination to a treatment decision (OF2). In addition, modern diagnostics increasingly require the use of MRI, however limited access and large variation in the use between the Norwegian breast diagnostic centres have been seen [27]. Because breast-conserving surgery provides a better prognosis than mastectomy a thorough examination of the breast is required to ensure removal of all tumour tissue [28]. Further the increasing use of oncoplastic procedures, reconstructions, and symmetrising interventions in the same surgery has increased surgical times, which has increased the demand on surgical rooms around the country.

### Prostate cancer

None of the health trusts met the national target that 70% of prostate cancer patients would receive surgery within 66 days. The increased waiting time can be explained by the widespread clinical practice of waiting at least six weeks after biopsy in order to reduce the possibility of significant inflammation and to improve the possibilities for nerve sparing prostatectomy. In addition, evidence also suggests that since prostate cancer treatment often have significant side effects such as incontinence, impotence, and decreased libido, patients request additional time to compare the pros and cons before making an informed decision. Attributable to prostate cancer patients’ relatively good prognosis with a long expected survival, recent studies found that non-metastatic prostate cancer patients with more than 180 days between diagnosis and surgery did not have adverse oncological outcomes or increased prostate-specific mortality, compared to patients who had surgery within 60 days of diagnosis [29, 30]. These findings raise the question if the time frames given in the CPPs for prostate cancer are clinically relevant or if they should be adjusted to account for the time the patient needs to make a decision.

This study has some limitations. First, the study period is the first two years after implementation of CPPs in Norway which may not reflect the current situation. While implementation issues related to IT systems, differing registration practices by hospital, and overall uncertainty around the whole concept of CPPs may have affected CPP waiting times, a dashboard published by the NPR shows no clear indication of a marked change in waiting times to treatment for any of the cancer types analysed in this paper [13]. Second, these data contain information about the first and not the main treatment given, which may result in an underestimation of some treatment modalities. For example, the proportion of prostate cancer patients treated with radiotherapy is known to be underestimated since neoadjuvant hormone therapy was categorised as SACT. Despite these limitations, the study’s population-based design and the use of national, comprehensive, high-quality data provide results that are widely representative. In addition, by using complete and detailed information, this study’s ability to examine each phase within the diagnostic and treatment pathway provides unique and valuable information about the waiting times of cancer patients.

## Conclusions

This study found that the recommended time frames of the overall pathway from referral to treatment were only met for colon and breast cancer patients who underwent surgery, and lung cancer patients who started SACT. A prolonged delay from treatment decision to start of treatment was the main reason why recommended time frames were not met. Cancer patients living in a health trust where their treatments were not provided experienced longer waiting time. Rectal and lung cancer patients were less likely to undergo radiotherapy and surgery, respectively, if they lived in a health trust that did not have a hospital performing the treatment. Clinicians and health administrators should identify ways of decreasing the prolonged waiting times to starting treatment possibly by increasing treatment capacity and should conscientiously accommodate expedited referrals of patients who must travel outside of their health trust for treatment. Compliance with recommended CPP guidelines should be examined further to determine if these time frames are still relevant and helpful for the patients, or if there is reason to update the recommendations or even the structure of the CPP itself.

## Supplementary Information

**Additional file 1:** **Supplementary Figure 1.** The cumulative proportion per risk group of prostate cancer patients diagnosed in 2015-2016 in Norway by OF1, OF2, OF3 and OF4.

**Additional file 2:** **Supplementary Table 1.** Definition of “Treatment hospital in health trust” for each health trust by surgery and radiotherapy and cancer type.

**Additional file 3:** **Supplementary Table 2.** Patient characteristics by part of the cancer patient pathway for colon cancer patients diagnosed in 2015–2016 in Norway.

**Additional file 4:** **Supplementary Table 3.** Patient characteristics by part of the cancer patient pathway for rectal cancer patients diagnosed in 2015–2016 in Norway.

**Additional file 5:** **Supplementary Table 4.** Patient characteristics by part of the cancer patient pathway for lung cancer patients diagnosed in 2015–2016 in Norway.

**Additional file 6:** **Supplementary Table 5.** Patient characteristics by part of the cancer patient pathway for breast cancer patients diagnosed in 2015–2016 in Norway.

**Additional file 7:** **Supplementary Table 6.** Patient characteristics by part of the cancer patient pathway for prostate cancer patients diagnosed in 2015-2016 in Norway.

**Additional file 8:** **Supplementary Table 7.** The proportion of OF1, OF2, OF3 and OF4 within the recommended time frames among colon cancer patients diagnosed in 2015–2016 in Norway.

**Additional file 9:** **Supplementary Table 8.** The proportion of OF1, OF2, OF3 and OF4 within the recommended time frames among rectal cancer patients diagnosed in 2015–2016 in Norway.

**Additional file 10:** **Supplementary Table 9.** The proportion of OF1, OF2, OF3 and OF4 within the recommended time frames among lung cancer patients diagnosed in 2015–2016 in Norway.

**Additional file 11:** **Supplementary Table 10.** The proportion of OF1, OF2, OF3 and OF4 within the recommended time frames among breast cancer patients diagnosed in 2015–2016 in Norway.

**Additional file 12:** **Supplementary Table 11.** The proportion of OF1, OF2, OF3 and OF4 within the recommended time frames among prostate cancer patients diagnosed in 2015–2016 in Norway.

**Additional file 13:** **Supplementary Table 12.** Univariable and multivariable quantile regression for OF4 for colon cancer patients diagnosed in 2015–2016 in Norway.

**Additional file 14:** **Supplementary Table 13.** Univariable and multivariable quantile regression for OF4 for rectal cancer patients diagnosed in 2015–2016 in Norway.

**Additional file 15:** **Supplementary Table 14.** Univariable and multivariable quantile regression for OF4 for lung cancer patients diagnosed in 2015–2016 in Norway.

**Additional file 16:** **Supplementary Table 15.** Univariable and multivariable quantile regression for OF4 for breast cancer patients diagnosed in 2015–2016 in Norway.

**Additional file 17:** **Supplementary Table 16.** Univariable and multivariable quantile regression for OF4 for prostate cancer patients diagnosed in 2015–2016 in Norway.

## Data Availability

The data that support the findings of this study are publicly available upon request from the Cancer Registry of Norway, The Norwegian Patient Registry and Statistics Norway.

## Abbreviations

CI: Confidence interval
CPP: Cancer patient pathway
CRN: Cancer Registry of Norway
EAU: European Association of Urology
ICD10: International Classification of Diseases 10^th^ edition
NPR: Norwegian Patient Registry
OF: Organ-specific pathway time indicator
SACT: Systemic anticancer treatment
SES: Socioeconomic status
