# Supplementary material for: Compliance with recommended cancer patient pathway timeframes and choice of treatment differed by cancer type and place of residence among cancer patients in Norway in 2015–2016
Source: BMC Cancer. 2022 Feb 28;22:220. doi: 10.1186/s12885-022-09306-9 (PMC8883731; doi:10.1186/s12885-022-09306-9)
Supplement: Supplementary file 1 — Additional file 1: Supplementary Figure 1. The cumulative proportion per risk group of prostate cancer patients diagnosed in 2015-2016 in Norway by OF1, OF2, OF3 and OF4. [file 12885_2022_9306_MOESM1_ESM.pdf]

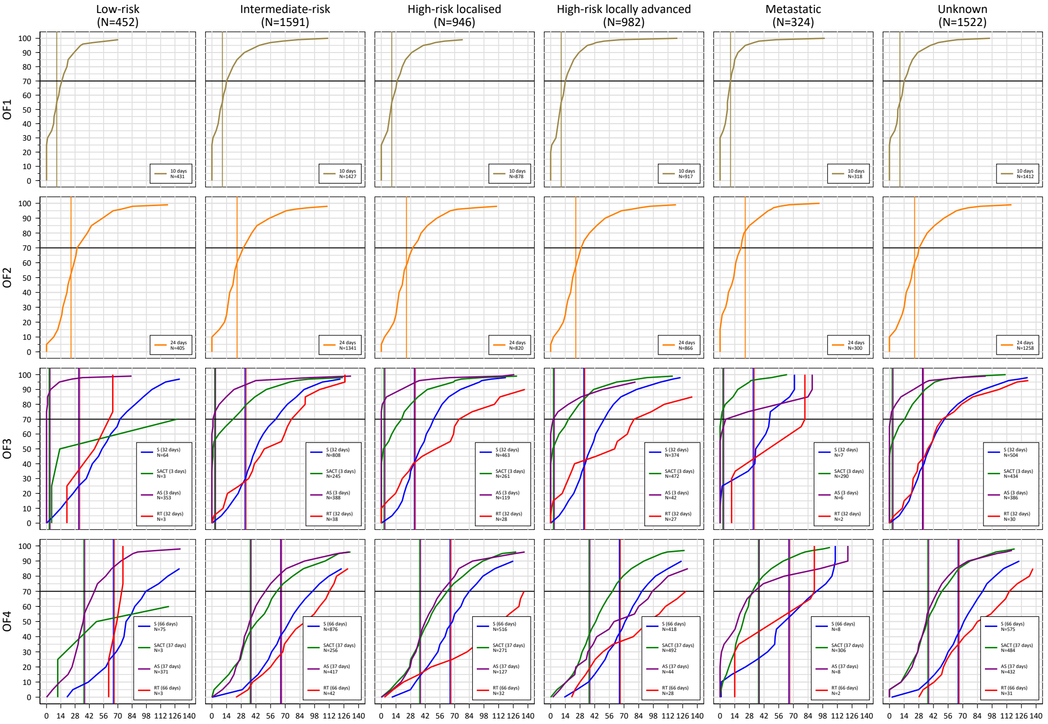

Supplementary Figure 1: The cumulative proportion per risk group of prostate cancer patients diagnosed in 2015-2016 in Norway by OF1, OF2, OF3 and OF4.
